# Diurnal Variations in Neural Activity of Healthy Human Brain Decoded with Resting-State Blood Oxygen Level Dependent fMRI

**DOI:** 10.3389/fnhum.2016.00634

**Published:** 2016-12-20

**Authors:** Chunxiang Jiang, Li Yi, Shi Su, Caiyun Shi, Xiaojing Long, Guoxi Xie, Lijuan Zhang

**Affiliations:** ^1^Paul C. Lauterbur Research Center for Biomedical Imaging, Shenzhen Institutes of Advanced Technology, Chinese Academy of SciencesShenzhen, China; ^2^Department of Neurology, Peking University Shenzhen HospitalShenzhen, China

**Keywords:** diurnal rhythm, fMRI, ReHo, ALFF, neural activity

## Abstract

It remains an ongoing investigation about how the neural activity alters with the diurnal rhythms in human brain. Resting-state functional magnetic resonance imaging (RS-fMRI) reflects spontaneous activities and/or the endogenous neurophysiological process of the human brain. In the present study, we applied the ReHo (regional homogeneity) and ALFF (amplitude of low frequency fluctuation) based on RS-fMRI to explore the regional differences in the spontaneous cerebral activities throughout the entire brain between the morning and evening sessions within a 24-h time cycle. Wide spread brain areas were found to exhibit diurnal variations, which may be attributed to the internal molecular systems regulated by clock genes, and the environmental factors including light-dark cycle, daily activities and homeostatic sleep drive. Notably, the diurnal variation of default mode network (DMN) suggests that there is an adaptation or compensation response within the subregions of DMN, implying a balance or a decoupling of regulation between these regions.

## Introduction

Circadian rhythm of sleep and wakefulness is a fundamental property of human physiology, which is important for sustaining essential body functions. It has been reported that the physiology of human brain is rhythmic genetically and systematically (Smale et al., 2008). The suprachiasmatic nuclei (SCN), located in a small region of the hypothalamus, is considered to be the primary circadian clock in mammals (Hastings et al., 2003; Saper et al., 2005). SCN activity fluctuates on a daily basis (Hofman and Swaab, 1993). Understanding system properties intrinsic to the SCN, as well as its regulation of peripheral oscillators throughout the brain and body will provide a fundamental contribution to our understanding of the neurophysiological basis of human circadian behavior.

The fluctuating activities of SCN and peripheral oscillators, in concert with the learning and experience over the course of a day may influence neurons activity and the way brain regions communicate with each other. Resting-state functional magnetic resonance imaging (RS-fMRI) technique can investigate brain activity by measuring the variance in the spontaneous fluctuations of the blood oxygen level dependent (BOLD) signal. Based on RS-fMRI, regional homogeneity (ReHo) and amplitude of low frequency fluctuation (ALFF) methods have been used to analyze spontaneous low-frequency (<0.08 Hz) blood oxygenation level-dependent fluctuations of the brain (Zang et al., 2004; Yang et al., 2007). ReHo measures the coherence of low frequency fluctuation within a given area, which can be used to assess the synchrony of neural activity based on the hypothesis that brain activities would more likely occur in clusters than in a single voxel. ALFF is associated with field potential activity of local brain regions (Logothetis et al., 2001) that could be employed to evaluate the intensity of intrinsic or spontaneous neuronal activity of the brain (Yu-Feng et al., 2007). Therefore, ReHo and ALFF can serve as sensitive markers indicating the alteration of brain function. For example, ReHo decreases in the posterior cingulate cortex/precuneus (PCC/PCu) in the AD patients (He et al., 2007), ALFF increases in the anterior portion of the dorsal anterior cingulate cortex in subthreshold depression patients (Li et al., 2014).

A recent research investigated if the brain networks are stable during a 24-h period by acquiring RS-fMRI data from 12 young adults at 3-h intervals over 24 consecutive hours. The authors found resting state sub-network dynamicity, especially the dynamic fluctuations of default mode network (DMN) connectivity and speculated that connectivity may have different spatiotemporal properties on the neural level (Park et al., 2012). Marek et al. (2010) also reported that the orienting and executive attention neuronal networks present time-of-day related variations with a stroop-like task performed five times a day. In our previous work, we have found a diurnal variation pattern of white matter microstructure which may function as the substrates of the phasic neural activities in correspondence to the environment adaptation in a light-dark cycle (Jiang et al., 2014). These analyses of the spontaneous neural activities in response to the diurnal rhythm may provide a new insight toward better understanding of the brain physiology and sleep medicine. Therefore, we aim to investigate the dynamic characteristic of neural activity by evaluating the intra-individual variability of ReHo and ALFF over one light-dark cycle.

## Materials and Methods

### Subjects

This study was approved by the institutional review board of Shenzhen Institutes of Advanced Technology. Informed consent was obtained from each of the 16 healthy subjects (6 males, 10 females, 23–31 years, mean age 24.8 ± 2.0 years) prior to the MRI examinations. All the subjects were graduate students whose daily activities follow a campus routine in which the regular meal time, bedtime (11:00 pm ± 1 h), and taking classes for the rest time were followed. No participant was excluded because of neurological or psychiatric illnesses, sleep disorder, drug, coffee, smoking, or alcohol abuse.

### MRI Data Acquisition

All the experiments were performed on a 3T Siemens MRI scanner (Siemens Trio system, Erlangen, Germany) with a 12-channel head coil in a scan room with temperature controlled between 23 to 24°C. High resolution T1 weighted whole brain scan was acquired using MPRAGE sequence for anatomical reference with TR/TE/TI 1900/2.53/900 ms, flip angle 9°, field of view (FOV) 250 mm, slice thickness 1 mm, acquisition matrix 256 × 256. Resting state BOLD images were acquired axially with an echo-planar imaging (EPI) sequence with TR/TE 3000/30 ms, flip angle 90°; FOV 210 mm, matrix 128 × 128, 30 slices, thickness 3 mm, bandwidth 1395 Hz/pixel, 60 volumes. Subjects were instructed to keep their eyes closed, relax their minds and remain motionless as much as possible but were requested not to fall asleep during the MRI data acquisition. Integrated parallel acquisition technique (iPAT) with acceleration factor of 2 was used to reduce the acquisition time and image distortion from susceptibility artifacts.

For each subject the MRI data was acquired in the morning at 8:30 am ± 0.5 h and repeated in the evening at 7:30 pm ± 0.5 h during a 24-h interval, about 0.5–1 h after meal before the MRI acquisition.

### Data Preprocessing

Data preprocessing was carried out using Data Processing Assistant for Resting-State fMRI (DPARSF^1^). The first 10 volumes of each time series were discarded for the instability of the initial MRI signal and subjects’ adaptation. The remained fMRI data were corrected for within-scan acquisition time differences between slices. After head motion corrected by realignment of all consecutive volumes to the first image [using a least square approach and a six parameter (rigid body) spatial transformation], the fMRI data were coregistered (rigid-body transformation) to T1-weighted images, and then spatially normalized (12-parameter affine transformation) to the MNI (Montreal Neurological Institute) space and resample to 3 mm × 3 mm × 3 mm. Temporal band-pass filtering (0.01–0.08 Hz) was then used to remove the linear trend of time courses to reduce low-frequency drift and physiological high frequency respiratory and cardiac noise. ReHo was defined by Kendall’s coefficient of concordance (KCC) which was used to measure ReHo of the time series of a given voxel with those of its nearest neighbors according to formula 1 (Zang et al., 2004). In order to reduce the effect of individual variability, ReHo was divided by the global mean value for each subject. Then the ReHo map was smoothed with a Gaussian filter of 4 mm full width at half-maximum (FWHM).

$$W=\frac{\Sigma R_{i}^{2}-{n\left(\overset{¯}{R}\right)}^{2}}{\frac{1}{12}K^{2}\left(n^{3}-n\right)}$$

where *W* is the KCC among given voxels, ranged from 0 to 1; *R*_i_ is the sum rank of the *i*th time point; where $\overset{¯}{R}=((n+1)K)/2$ is the mean of the *R*_i_’s; *K* is the number of time series within a measured cluster (27, one given voxel plus the number of its neighbors); *n* is the number of ranks (here *n* = 50).

The filtered time series were converted to a frequency domain using a Fast Fourier Transform. Then the square root of power spectrum was calculated and averaged within the frequency range between 0.01 and 0.08 Hz at each voxel. ALFF was defined by the averaged square root. In order to reduce the effect of variability across participants, the ALFF of each voxel was divided by the global mean ALFF value. A more detailed methodology of ALFF can be found in previous studies (Yang et al., 2007; Yu-Feng et al., 2007).

### Statistical Analysis

A one sample *t*-test was performed within each scan session to explore which brain regions show higher ReHo/ALFF than global mean value (*p* < 0.001, FDR corrected). Paired *t*-test was applied to compare the ReHo/ALFF difference between the morning and evening scan sessions. Voxels with *p* < 0.05 and cluster size >85 voxels were considered to be statistically significant (corrected by AlphaSim program). Two statistic parametric files were obtained during the paired *t*-test in ReHo and ALFF, which were then used to extract the overlapped brain regions in ReHo and ALFF by a homemade program implemented in MATLAB (Mathworks, Natick, MA, USA). Parametric data was mapped to the T1 weighted images to facilitate visualization.

## Results

### Diurnal Variations of ReHo

**FIGURE 1:**
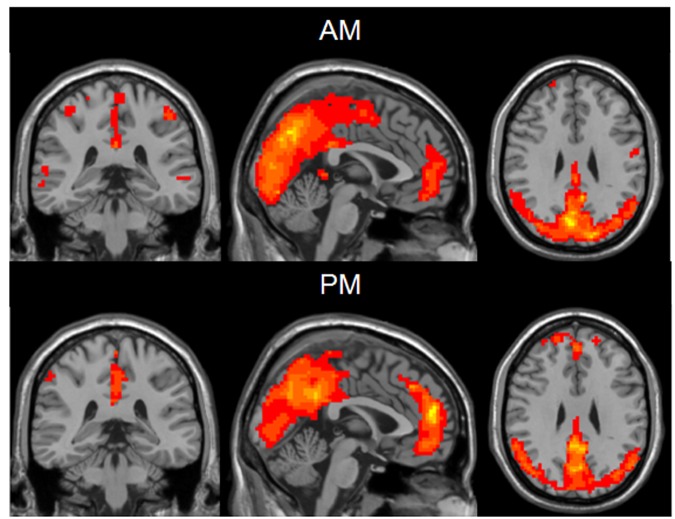
**One sample *t*-test results of mean ReHo maps within the AM group **(Top)** and PM group **(Bottom)** (MNI coordinates *x* = -1, *y* = -35, *z* = 29).** Statistical maps were overlapped on the anatomical template for visualization.

The results of inner-group ReHo analysis were shown in **Figure 1**. Voxels in the middle occipital gyrus and the DMN including PCC, PCu, medial prefrontal cortex (MPFC) and bilateral inferior parietal lobe (IPL) exhibited significantly higher ReHo relative to the rest brain areas. The neural activity of the AM and PM sessions were comparable in pattern but different in strength.

**FIGURE 2:**
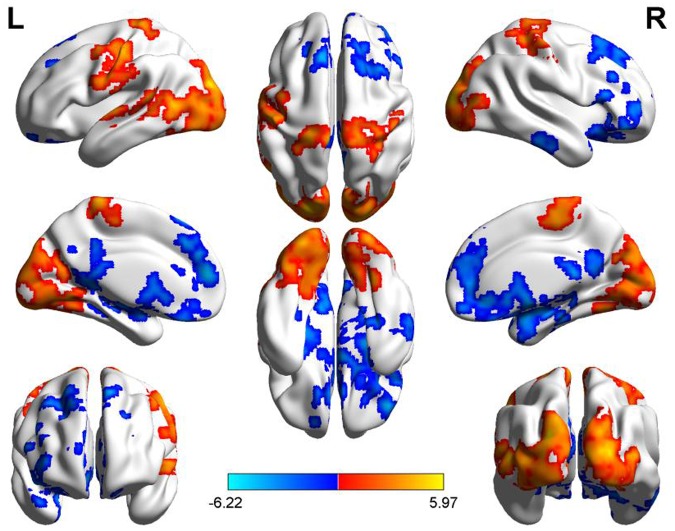
**Diurnal changes of ReHo.** The final statistical maps are visualized by eight views (The first row from left to right is lateral view of left hemisphere, top side, lateral view of right hemisphere. The second row from left to right is medial view of left hemisphere, bottom side, medial view of right hemisphere. The third row is frontal and back side). Color bar indicates *t*-value (Red–yellow, AM > PM, blue–green, AM < PM).

Compared to the PM session, the AM data showed a significant ReHo increase in the areas including bilateral middle and inferior occipital gyrus (BA17, BA18, BA19), lingual gyrus, fusiform gyrus, precentral and postcentral gyrus, cuneus and paracentral lobule, left middle and superior temporal gyrus and decreased in bilateral superior and middle frontal gyrus, medial- and inferior-orbital frontal gyrus, superior frontal gyrus, anterior cingulate, PCC, PCu, parahippocampal gyrus, hippocampus, caudate, amygdala and right inferior-orbital frontal gyrus (**Figure 2**) (summarized in **Tables 1** and **2**). In summary, the ReHo mainly increased in the morning session in the occipital, parietal and left temporal lobes, and decreased in frontal and anterior-posterior cingulate midline.

**Table 1:** Summary of brain regions with significant increased ReHo in AM group compared to PM group.

Brain regions	Peak coordinate	Cluster size	Maximum *t-*value
Cuneus_R	(21 -96 12)	187	5.9733
Occipital_Mid_R		116	
Lingual_R		172	
Occipital_Sup_R		190	
Occipital_Inf_R		54	
Fusiform_R		50	
Calcarine_R		140	
Postcentral_R		297	
Precentral_R		71	
Paracentral_Lobule_R		78	
Cuneus_L		167	
Occipital_Mid_L		336	
Lingual_L		325	
Occipital_Sup_L		228	
Occipital_Inf_L		142	
Fusiform_L		117	
Calcarine_L		189	
Postcentral_L		203	
Precentral_L		92	
Paracentral_Lobule_L	(-6 -36 72)	82	4.636
Temporal_Mid_L		262	
Temporal_Sup_L	(-54 -6 0)	85	4.3854


**Table 2:** Summary of brain regions with significant decreased ReHo in AM group compared to PM group.

Brain regions	Peak coordinate	Cluster size	Maximum *t*-value
Frontal_Sup_Orb_R	(18 21 -15)	27	-5.887
Frontal_Mid_Orb_R		31	
Frontal_Sup_R		128	
Frontal_Mid_R		117	
Frontal_Sup_Medial_R		111	
Frontal_Inf_Orb_R		96	
ParaHippocampal_R		111	
Hippocampus_R		96	
Amygdala_R		36	
Precuneus_R	(-21 -39 27)	100	-6.2167
Cingulum_Ant_R		102	
Cingulum_Post_R		32	
Caudate_R		14	
Frontal_Sup_Medial_L		234	
Frontal_Sup_L		65	
Frontal_Sup_Orb_L		17	
Frontal_Med_Orb_L		20	
Frontal_Mid_L		15	
ParaHippocampal_L		32	
Hippocampus_L		49	
Amygdala_L	(-27 -3 -15)	26	-4.173
Precuneus_L		60	
Cingulum_Ant_L		41	
Cingulum_Post_L		28	
Caudate_L		64	


### Diurnal Changes of ALFF

**FIGURE 3:**
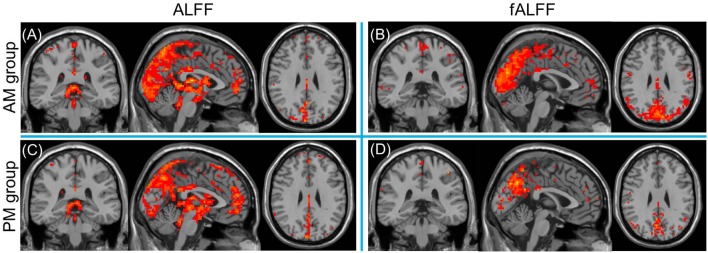
**One sample *t*-test results of mean ALFF** (**A,C**, left column) and fALFF (**B,D**, right column) maps within the AM group (top row) and PM group (bottom row) (MNI coordinates *x* = -1, *y* = -35, *z* = 29). Statistical maps were overlapped on the anatomical template for visualization.

For the inner-group ALFF analysis, significant increased ALFF was shown in PCC, MPFC, PCu, and the non-specific areas including ventricles and cisterns (**Figures 3A,C**) within AM and PM sessions. As ALFF is sensitive to physiological noise irrelevant to brain activity (Zou et al., 2008), fractional ALFF (fALFF) method, a ratio of the power of each frequency at the low-frequency range (0.01–0.08 Hz) to that of the entire frequency range, was applied to suppress the non-specific signal components in the rs-fMRI and improve the sensitivity in detecting regional spontaneous brain activity. Higher fALFF was found in bilateral occipital lobes, PCC, PCu, cuneus, IPL, MPFC, middle and superior temporal gyrus, precentral and postcentral gyrus, as shown in **Figures 3B,D**.

**FIGURE 4:**
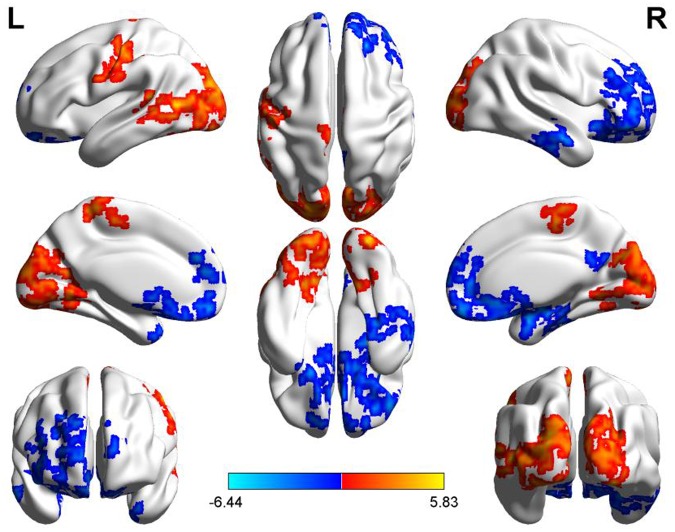
**Diurnal changes of ALFF.** The final statistical maps are visualized by eight views (The first row from left to right is lateral view of left hemisphere, top side, lateral view of right hemisphere. The second row from left to right is medial view of left hemisphere, bottom side, medial view of right hemisphere. The third row is frontal and back side). Color bar indicates *t*-value (Red–yellow, AM > PM, blue–green, AM < PM).

Compared to the PM session, significant increased morning ALFF occurred in left precentral and postcentral gyrus, middle and superior temporal gyrus, bilateral paracentral lobule, lingual gyrus, cuneus, superior and middle and inferior occipital gyrus, and decreased in the area including bilateral anterior cingulate, rectus, medial- and inferior-orbital frontal gyrus, superior frontal gyrus, and right posterior cingulate, amygdala, hippocampus, parahippocampal gyrus, PCu, middle and inferior temporal gyrus (**Figure 4**) (summarized in **Tables 3** and **4**).

**Table 3:** Summary of brain regions with significant increased ALFF in AM group compared to PM group.

Brain regions	Peak coordinate	Cluster size	Maximum *t*-value
Lingual_L	(-18 -75 -12)	160	5.8333
Occipital_Mid_L		250	
Occipital_Sup_L		131	
Occipital_Inf_L		50	
Calcarine_L		162	
Cuneus_L		123	
Temporal_Mid_L		153	
Postcentral_L	(-51 -18 51)	89	4.5896
Precentral_L		23	
Paracentral_Lobule_L		47	
Occipital_Sup_R		74	
Occipital_Mid_R		68	
Occipital_Inf_R		17	
Cuneus_R		134	
Lingual_R		66	
Paracentral_Lobule_R	(6 -27 66)	16	3.977


**Table 4:** Summary of brain regions with significant decreased ALFF in AM group compared to PM group.

Brain regions	Peak coordinate	Cluster size	Maximum *t*-value
Amygdala_R	(24 0 -21)	24	-6.4419
ParaHippocampal_R		64	
Hippocampus_R		56	
Fusiform_R		30	
Precuneus_R		26	
Frontal_Sup_R		94	
Frontal_Mid_R		142	
Frontal_Inf_Orb_R		86	
Frontal_Sup_Medial_R		87	
Rectus_R	(3 54 -15)	77	-6.2089
Cingulum_Ant_R		36	
Cingulum_Post_R		20	
Temporal_Inf_R	(51 -33 -21)	44	-4.3871
Temporal_Mid_R		28	
Frontal_Sup_L		33	
Frontal_Med_Orb_L		31	
Frontal_Inf_Orb_L		26	
Cingulum_Ant_L		20	
Rectus_L		72	


### Brain Areas with Both ReHo and ALFF Variations

**FIGURE 5:**
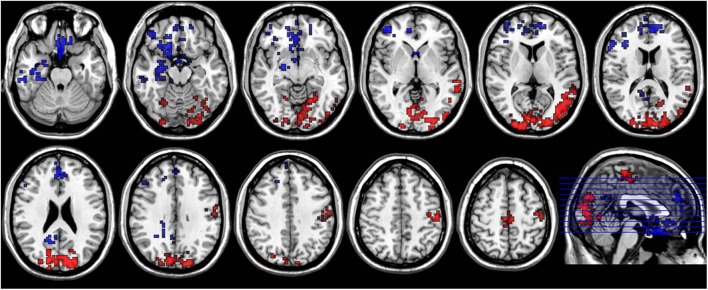
**Axial sections show areas with significant diurnal variations of both ReHo and ALFF.** The left hand side of the image is the right side of the brain (radiology convention). The hot color indicates increased ReHo and ALFF in AM compared to PM, and cool color indicates decreased ReHo and ALFF.

Compared to the PM session, ReHo and ALFF of the morning session were both increased in bilateral middle and inferior occipital gyrus (BA17, BA18, BA19), lingual gyrus, cuneus, paracentral lobule, left precentral and postcentral gyrus, left middle and superior temporal gyrus, and decreased in bilateral anterior cingulate, superior frontal gyrus, rectus, medial- and inferior-orbital frontal gyrus, amygdala, right PCC, inferior temporal gyrus and parahippocampal gyrus (**Figure 5**).

## Discussion

The current study demonstrated the diurnal rhythmic dynamic of the neural activity of human brain. Circadian rhythms are widespread in human brain and influenced by the interactions between internal molecular systems and environmental cues (Cirelli, 2009; Mohawk et al., 2012). Core clock genes are known to comprise transcriptional-translational auto-regulatory complexes. These genes make up a group of auto-regulatory loops with diurnal variation of activity and present rhythmic expression of their own and their regulatory target transcrips. Li et al. (2013) reported that there exists a rhythmic rise and fall in the transcriptional activity of hundreds of genes [including three Period homolog (PER 1-2-3) genes; brain and muscle Arnt-like protein-1 (BMSL1) and so on] initiating or responding to the regulation of 24-h behavioral and hormonal cycles. Vicentic et al. (2005) also found that CART (cocaine- and amphetamine-regulated transcript) peptides exhibit a diurnal rhythm in several brain regions including nucleus accumbens, hypothalamus and amygdala. Muto et al. (2016) investigated the local modulation of human brain responses by circadian rhythmicity and sleep debt. They found that the response of some subcortical regions like thalamus, head of caudate nucleus and putamen follows a 24 h circadian rhythmicity, and some brain regions were affected significantly by sleep debt, and some are affected by the interaction of the two factors (Muto et al., 2016). The circadian rhythmicity may substrate the physiological mechanisms of the diurnal variation of ReHo and amplitude fluctuation of neural activity occurred in some of the structures mentioned above in the current study.

The DMN is supposed to be involved in self-referential functions such as internal mentation, recollection and imagination and conceptual processing, containing a set of interacting brain areas that are functionally connected. It can be divided into sub-networks contributing to specific tasks or intrinsic cognitive demand (Mayer et al., 2010; van Buuren et al., 2010). The current study demonstrated that overall the DMN is involved in greater neural activity at resting state in both morning and the evening as compared to the other brain areas, which is consistent with previous findings (Raichle et al., 2001; Fransson, 2005). But some of the structures were found to be more subject to the diurnal alterations at the neural activity level, such as MPFC, medial temporal lobe, PCu and PCC. The divergence of the diurnal variation in these structures may suggest the functional coordination of the anatomically independent gyri. It has been reported that perturbations in DMN activity during wakefulness display co-occurring abnormalities of sleep in a number of disorders (Cherkassky et al., 2006; Garrity et al., 2007; Zhao et al., 2007). The DMN functional connectivity was found to be reduced in sleep deprivation (De Havas et al., 2012). The PCu and PCC, which demonstrated increased activity in the PM group, play a pivotal role in the mediation of intrinsic activity through DMN (Fransson and Marrelec, 2008). In the contrast, the activity of medial temporal lobe decreased in the PM group. Considered in the context of these findings, it’s reasonable to hypothesize that diurnal changes in DMN activity reflect adaptation or compensation response under continued wakeful condition. It may also imply that there is a balance of neural activity within DMN subregions, or a decoupling of regulation between these regions.

The occipital lobe shows significant decrease in ReHo and ALFF map in the PM group than that in the AM group. These findings are contrary to our initial assumption. The occipital lobe is mainly related to the visual information processing. The photic stimulus duration has an effect of “temporal integration” on neurons (Dkhissi-Benyahya et al., 2000), which means the neural activity should increase in the PM group. Also, decreased ADC (apparent diffusion coefficient) mainly occurred in the occipital lobe in the PM group in our previous study on diurnal variation of white matter using diffusion tensor imaging (DTI), which is believed to associate with the increased neuron activation (Jiang et al., 2014). But Buysse Daniel et al. (2004) measured relative glucose metabolism in the morning and in the evening with PET, and found significantly lower glucose metabolism in the evening than in the morning in occipital lobe, including cuneus, medial occipital gyrus, lingual gyrus, and occipitotemporal gyrus, which was interpreted to reflect increasing homeostatic sleep drive and/or the restorative effect of sleep. Muto et al. (2016) reported that the neural response of occipital gyrus are modulated by the interaction of circadian rhythmicity and sleep debt. These investigations suggest that sleep debt may have dominant control in the occipital lobe in the evening under current experiment condition.

Precentral and postcentral gyri have higher homogeneous neural activity and low frequency fluctuation in the morning than in the evening. This may suggest that during the evening hours, the somatosensory and motor functions represent a weakened response to the afferent and/or efferent signals. The exact biological mechanisms behind ReHo and ALFF remain unclear up to date. ReHo was defined as the temporal homogeneity of the regional BOLD signal (Zang et al., 2004), while ALFF reflects the amplitude of low-frequency fluctuation in the range of 0.01–0.08 Hz (Yu-Feng et al., 2007). Logothetis and Wandell (2004) reviewed that the hemodynamic response primarily reflects the neuronal input to the relevant area of the brain and synaptic potentials are the strongest cause of the BOLD response. The lower ReHo and ALFF in precentral and postcentral gyri found in this study may indicate that the synchronization of neural activity in these areas is disrupted and the amplitude of low-frequency fluctuation of BOLD signal decreases, possibly corresponding to the deteriorated somatosensory and motor response to the external stimulus and weakened local field potential as a result of increased level of exhaustiveness, sleepiness, and absentmindedness after a day experience.

The prefrontal cortex (including medial- and inferior-orbital frontal gyrus, and anterior cingulate) shows greater cerebral BOLD response in the PM group than in the AM group. These regions are reported to be involved in alertness, attention and higher-order cognition processes. However, Thomas et al. (2000) found that brain activity and function decrease in prefrontal cortex and thalamus after 24 h of sleep deprivation using positron emission tomography (PET), which implies that the need for recuperation in these areas is greater than other brain regions. On the contrary, Drummond and Brown (2001) reported that prefrontal cortex and parietal lobes showed increased activation in verbal learning and divided attention tasks after total sleep deprivation using fMRI, which may suggest a specific compensatory mechanism designed to combat increased sleepiness. Increased evening activity in the prefrontal cortex was also identified in this study. We hypothesize that the increased sleepiness or fatigue after a day of activity may induce neurochemical consequences for the brain to adapt to fulfillment the demand for the alertness as well as the higher cognitive performance as dark cycle approaches.

## Limitations of Current Study

It should be noted that there are several limitations in this study. For example, the resting state fMRI signal is partially affected by cardiac pulsation, respiration, vasoconstriction, instrumental and thermal sources of noise. We didn’t record the physiological parameters mentioned above during the fMRI data acquisition. During the data preprocessing procedure, a temporal band-pass filter was used to reduce low frequency drift and high frequency physiological noise. This may alleviate, although not completely eliminate the effect on accuracy and reliability for the current study.

Sex related difference in cerebral structure and function was not taken into account in this study, which may confound the interpretation of the results. Evidences have been accumulating that sex disparity matters in the circadian modulation of mammal brain (Mong et al., 2011). Women are found to have shorter intrinsic periods and earlier phase of melatonin rhythms as well as greater night-time impairment in cognitive performance as compared to men (Duffy et al., 2011; Santhi et al., 2016), but the intrinsic circadian periods of both gender are close to 24 h. In addition, healthy people have different preferences in sleep pattern such as morning “lark” or evening “owl” chronotypes. This may also embed complicated mechanisms of rhythmic dynamics of human brain that this study did not reach.

Lastly, the fMRI data were acquired from only 16 young subjects at two time points. The sample size was relatively small. This can’t and limit us in providing more significant findings. A larger sample size covering multiple age groups and time points are expected to provide stronger statistical power with reduced individual effects and enable a more detailed tracking of neural activities during the time course of a day–night cycle.

Further study is needed to optimize the experiment design, like recruitment of single gender subjects, melatonin assays to assess circadian phase, performing different cognitive tasks that are differentially affected by sleep drive and circadian rhythmicity.

## Conclusion

Neural activity of healthy young human brain exhibits a diurnal pattern which may be related to circadian plasticity or accumulated waking, which may substrate the neurobiological and behavioral mechanisms of sleep hygiene and sleep disorders. Factors of time of day should be taken into account in the functional neuroimaging based neural studies even under resting conditions.

## Author Contributions

Conceived and designed the experiments: LZ and CJ. Performed the experiments: CJ, LY, and LZ. Analyzed the data: CJ, LY, SS, CS, XL, GX, and LZ. Wrote the paper: CJ and LZ.

## Conflict of Interest Statement

The authors declare that the research was conducted in the absence of any commercial or financial relationships that could be construed as a potential conflict of interest.

## Footnotes

1 http://www.restfmri.net
